# Risk factors of carotid plaque and carotid common artery intima‐media thickening in a high‐stroke‐risk population

**DOI:** 10.1002/brb3.847

**Published:** 2017-10-12

**Authors:** ChunFang Wang, GaoPeng Lv, DaWei Zang

**Affiliations:** ^1^ First Central Clinical College of Tianjin Medical University Tianjin China; ^2^ Department of Neurology Tianjin First Central Hospital Tianjin China; ^3^ Department of Neurology Tianjin First Central Hospital Tianjin University of Traditional Chinese Medicine Tianjin China; ^4^ Department of Neurology Tianjin First Central Hospital Tianjin Medical University Tianjin China

**Keywords:** carotid common artery intima‐media thickening, carotid plaque, high‐stroke‐risk population, risk factors

## Abstract

**Introduction:**

To analyze the risk factors of carotid plaque (CP) and carotid common artery intima‐media thickening (CCAIMT) and the association between the risk factors and CP numbers and the side of the CCAIMT in a high‐stroke‐risk population.

**Methods:**

Carotid ultrasonography was conducted in 2025 participants with high stroke risk. Participants were divided into different groups according to the results of the ultrasound. The risk factors and blood biochemical indices were recorded.

**Results:**

The presence of CP and CCAIMT were 38.9% and 24.8% respectively. Multivariate logistic regression indicated that the risk factors of CP were age, high LDL‐C and FBG levels, male gender, stroke, diabetes, hypertension, and tobacco use. Compared with participants without CPs, the participants who were male, and older in age, with risk factors of tobacco use, diabetes, high LDL‐C levels, and a family history of hypertension were likely to have a single CP, whereas the participants with risk factors of tobacco use, diabetes, hypertension, male gender, older age, high LDL‐C levels, stroke and AF or valvulopathy were prone to have multiple CPs. The risk factors of CCAIMT were male gender, stroke, hypertension, diabetes, AF or valvulopathy, tobacco use and age. Compared with the N‐CCAIMT subgroup, the risk factors of left CCAIMT were tobacco use, diabetes, male gender, and age. The risk factors of right CCAIMT were male gender, high FBG levels, age, AF or valvulopathy. The risk factors of dual CCAIMT were high frequency of drinking milk, tobacco use, male gender, age, stroke, and hypertension.

**Conclusion:**

These findings revealed the risk factors of CP and CCAIMT, and an association between the risk factors and the CP numbers and the side of the CCAIMT.

## 1 INTRODUCTION

Carotid atherosclerotic disease is recognized as an important risk factor for cerebral ischemic events. Carotid intima‐media thickening (CIMT) and carotid plaque (CP) are considered to be surrogate subclinical markers of early atherosclerosis, are associated with stroke risk (Georgios et al., 2006; Rundek et al., 2015), and can predict the occurrence of ischemic cerebrovascular events (Prati et al., 2008). There is a study that found that increased common carotid artery stiffness is associated with ischemic stroke (Tsivgoulis et al., 2006). Also, there are already some studies about the risk factors of CPs or CIMT, for example, a study found that elevated resting heart rate is associated with an increased odds of CIMT and the presence of CPs (Wang, Zhang, Sun, Wang, & Cao, 2016). Another study revealed that when compared with nonsmoker, both former and current smokers have a greater risk of CIMT and CPs (Hisamatsu et al., 2016). There are also associations between dyslipidemia and CP; a cross‐sectional study revealed that TG is an independent predictor of CP risk (Mi et al., 2016), and another study showed that combined LDL‐C and HDL‐C levels can predict the presence of CPs, and the LDL‐C/HDL‐C ratio can act as an independent index for CIMT (Yang et al., 2014). There are also associations between different obesity subtypes and carotid atherosclerosis; compared with metabolically healthy and normal‐weight subtypes, metabolically abnormal and obese subtypes have a positive association with CP and a higher value of CIMT (Kim, Kim, Eun, & Song, 2015). A study even revealed that prehypertension status is associated with carotid common artery intima‐media thickening (CCAIMT), prehypertensive patients have higher CCAIMT (Manios et al., 2009).

The development of atherosclerosis begins at an early age and lasts approximately 50 years; some risk factors, such as hypertension, diabetes, obesity, smoking, and genetic predisposition, can provoke or intensify the development of atherosclerosis (Insull, 2009). Atherosclerotic lesions do not have any symptoms in the early stage, but there would be serious consequences when the plaques rupture. Therefore, primary prevention and detection of atherosclerosis as well as modifications of the risk factors are quite important. Carotid ultrasound can detect the degree of carotid atherosclerosis by measuring CIMT and CP (Lee & Park, 2014).

Although there are studies that have analyzed the presence of CIMT and CPs in different populations (Ghouri et al., 2015; Giannopoulos et al., 2013; Hogberg, Kragsterman, Bjorck, Tjarnstrom, & Wanhainen, 2014; Zhan et al., 2016), studies about the number of CPs and the side of the CCAIMT are scarce, and the data on the risk factors of CP and CCAIMT in a high‐stroke‐risk population are also limited. In the present study, we aim to explore the characteristics of CP and CCAIMT risk factors among a high‐stroke‐risk population.

## 2 MATERIALS AND METHODS

### 2.1 Study design and population

This study was approved by The Clinical Experimentation Committee of Human of Tianjin First Central Hospital (2012N0049KY). All participants in the study signed an informed consent prior to enrollment. The study was performed between January 2013 and December 2015 in a study population from the Stoke Screening and Prevention Engineering of National Health and Family Planning Commission of the People's Republic of China. The total population are from two districts (one is an urban district, and the other one is a rural district) including 10924 residents, all of which are aged ≥40 years. After a primary screening of the demographic information, previous disease history, family history (FH), and other cardiovascular and cerebrovascular disease risk factors, 2025 residents (age range 40–97, 862 men, 1163 women) who had at least three risk factors or a previous history of transient ischemic attack (TIA) or stroke were enrolled and received carotid ultrasonography examination. The risk factors were as follows: (1) hypertension (HT); (2) atrial fibrillation (AF) or valvulopathy (val.); (3) tobacco use; (4) hyperlipidemia (HLP); (5) diabetes; (6) lack of physical exercise (PE); (7) overweight or obesity; (8) family history of stroke.

### 2.2 Survey procedure

After the primary screening for all of the residents, a high‐risk group of 2025 participants was determined. Local doctors informed the high‐risk participants to fast overnight before the examination. On the examination day, the trained researchers completed a more‐detailed risk factor survey via questionnaires and performed physical examinations. Blood samples were collected by nurses at the local health center. All blood samples were sent to the laboratory of the Tianjin First Central Hospital for analysis. Measurements of CPs and CCAIMT were performed by experienced technicians. A 12‐lead echocardiogram was also performed at the same time.

### 2.3 Physical examinations

Blood pressure (BP), height, weight, waistline, hipline, and pulse were measured. Body mass index (BMI) was calculated as the weight (kg) divided by the square of height (m^2^). Waist‐hip ratio (WHR) was calculated as the waistline (m) divided by the hipline (m). Serum fasting blood glucose (FBG), total cholesterol (TC), total triglyceride (TG), high‐density lipoprotein cholesterol (HDL‐C), and low‐density lipoprotein cholesterol (LDL‐C) levels were measured and analyzed at the laboratory of the Tianjin First Central Hospital. Carotid ultrasonography and 12‐lead echocardiography were also performed.

### 2.4 The survey for risk factors

The surveys were conducted through face‐to‐face interviews by trained researchers to collect the following data: name, gender, date of birth, educational level, previous history of hypertension, hyperlipidemia, diabetes, stroke, transient ischemic attack, atrial fibrillation, or valvulopathy, as well as family history of hypertension, diabetes, hyperlipidemia, stroke, and coronary heart disease (CHD), overweight or obesity, tobacco use, alcohol use, life styles including physical exercise, and the consumption of vegetables, fruits, and milk. The criteria of risk factors was listed in Table 1 (Joint Committee for Developing Chinese guidelines on Prevention and Treatment of Dyslipidemia in Adults, 2007; Zhou, 2002). The use of antihypertensive, lipid‐lowing, and glucose‐lowing medications within the past 2 weeks before the survey was also self‐reported.

**Table 1:** The criteria of risk factors

Risk factors	Criteria
Hypertension	Systolic BP (SBP)≥140 mmHg or diastolic BP (DBP)≥90 mmHg or taking medication for hypertension
Hyperlipidemia	TC≥6.22 mmol/L,TG≥2.26 mmol/L,LDL‐C ≥ 4.14 mmol/L or HDL‐C<1.04 mmol/L (Zhan et al., 2016)
Diabetes	FBG≥7.0 mmol/L or taking medication for diabetes
Overweight	BMI of 24.0–27.9 kg/m^2^ (Ghouri et al., 2015)
Obesity	BMI≥28.0 kg/m^2^ (Ghouri et al., 2015)
Tobacco use	≥1 cigarette per day for≥1 year
Alcohol use	Drinking alcohol≥1 time per week for 1 year
Physical exercise	≥3 times per week and >30 mins each time or heavy physical labor
Eat vegetables	≥5 days per week
Eat fruits	≥3 days per week
Drink milk	≥200 ml everyday and 5 days per week

### 2.5 Ultrasonography measurements

Practiced technicians performed the ultrasound exams. The patients were examined in the supine position by B‐mode ultrasonography (Mindray M7, Shenzhen, China) with a 7L4S linear array probe. The common carotid artery, internal carotid artery, subclavian artery and vertebral artery on dual sides were screened for plaque and intima‐media thickening. Plaques are defined as focal structures that encroach into the arterial lumen by at least 0.5 mm or 50% of its surroundings. IMT is defined as a thickness of >1.0 mm, as measured from the intima‐lumen interface to the media adventitia interface (Touboul et al., 2012).

### 2.6 Statistical analysis

Statistical analysis was performed by the IBM SPSS Statistics software (version 22.0). The normality distributions of continuous variables were tested by Kolmogorov–Smirnov tests. Continuous variables were presented as the mean ± standard deviations (*SD*). Categorical variables were presented as frequencies and percentages. The results of the logistic regression analyses were presented as odds ratios (OR) and 95% confidence intervals (CI). Student's *t* test or analysis of variance (ANOVA) were used to analyze nonpaired samples and for the comparison of normally distributed parameters. The Chi‐squared test was used for the comparison of categorical variables. We also applied univariate and multivariate logistic regression analyses to analyze the association between the positive results of the carotid ultrasonography and cerebrovascular risk factors. The study population was divided into different groups in order to perform multivariate logistic regression analyses. Risk factors with *p *<* *.1 in the univariate analyses and had clinical significance were selected as covariates in the multivariate models. *p *<* *.05 in the two‐sided tests were considered significant.

## 3 RESULTS

In total, 2025 residents were registered in this study, including 862 (42.6%) men and 1163 (57.4%) women. The mean age of the participants was 61.5 ± 9.9 years and ranged from 40 to 97 years.

**Figure 1:**
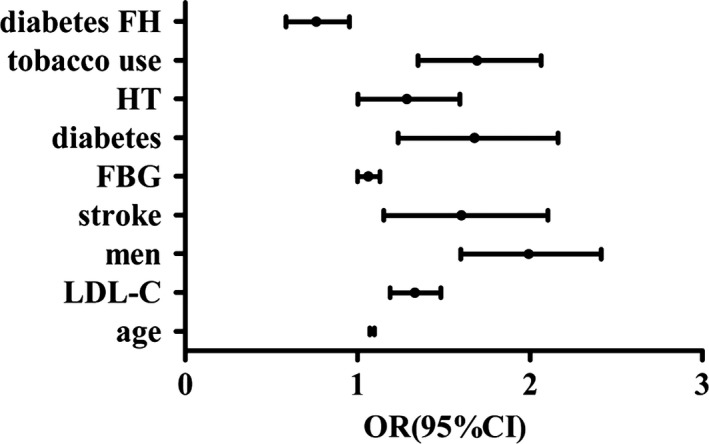
The risk factors of CP. Figure 1 shows the risk factors of CP are age, high LDL‐C level, male gender, stroke, high FBG level, diabetes, hypertension, tobacco use, but family history of diabetes is protective

First, all of the participants were divided by the presence of CPs. The presence of CPs was 52.9% in men and 47.1% in women (*p *<* *.05). The age, FBG, TC, LDL‐C levels and the presence of stroke, hypertension, hyperlipidemia, diabetes, AF or valvulopathy, tobacco use, alcohol use, and physical exercise of the CP group were significantly higher than the N‐CP group. While the presence of overweight or obesity, family history of CHD, hypertension, diabetes, and the frequency of eating vegetables and fruits of the N‐CP group were higher than the CP group (all *p *<* *.05; Table 2).The multivariate logistic regression analysis indicated that age, high LDL‐C and FBG levels, male gender, stroke, diabetes, hypertension, and tobacco use were independent risk factors of CP. A family history of diabetes was associated with a lower risk of CPs (Table 3, Figure 1).

**Table 2:** Demographic characteristics of the participants, based on the presence of CP

	CP	N‐CP
Total, *n* (%)	787 (38.9)	1238 (61.1)
Age (years)	65.9 ± 8.7*	58.7 ± 9.7
BMI (kg/m^2^)	25.4 ± 3.4	25.6 ± 3.8
WHR	0.9 ± 0.1	0.9 ± 0.1
FBG (mmol/L)	6.1 ± 2.1*	5.7 ± 1.6
TG (mmol/L)	2.0 ± 1.3	2.0 ± 1.4
TC (mmol/L)	5.0 ± 1.3*	4.8 ± 1.2
HDL‐C (mmol/L)	1.5 ± 0.6	1.5 ± 0.5
LDL‐C (mmol/L)	2.9 ± 1.1*	2.7 ± 0.9
Men (%)	416 (52.9)*	446 (36.0)
Women (%)	371 (47.1)*	792 (64.0)
Stroke (%)	122 (15.5)*	112 (9.0)
TIA (%)	97 (12.3)	159 (12.8)
HT (%)	611 (77.6)*	881 (71.2)
HLP (%)	570 (72.4)*	809 (65.3)
Diabetes (%)	204 (25.9)*	209 (16.9)
AF/val. (%)	138 (17.5)*	159 (12.8)
Tobacco use (%)	340 (43.2)*	390 (31.5)
Alcohol use (%)	223 (28.3)*	281 (22.7)
PE (%)	393 (49.9)*	562 (45.4)
Overweight/obesity (%)	309 (39.3)*	548 (44.3)
Stroke FH (%)	279 (35.5)	478 (38.6)
CHD FH (%)	238 (30.2)*	451 (36.4)
HT FH (%)	441 (56.0)*	766 (61.9)
Diabetes FH (%)	163 (20.7)*	342 (27.6)
HLP FH (%)	174 (22.1)	310 (25.0)
Vegetables (%)	648 (82.3)*	1075 (86.8)
Fruits (%)	383 (48.7)*	690 (55.7)
Milk (%)	171 (21.7)	281 (22.7)

*p <* *.05 versus N‐CP group.

BMI, body mass index; WHR, waist‐hip ratio; TC, total cholesterol; TG, total triglyceride; HDL‐C, high‐density lipoprotein cholesterol; LDL‐C, low‐density lipoprotein cholesterol; HLP, hyperlipidemia; PE, physical exercise; FH, family history

**Table 3:** The risk factors of CP

	OR	OR (95% CI)	*p*
Age	1.083	1.071–1.096	.000
LDL‐C	1.327	1.188–1.484	.000
Men	1.964	1.598–2.415	.000
Stroke	1.555	1.150–2.103	.004
FBG	1.062	1.000–1.128	.048
Diabetes	1.635	1.235–2.163	.001
HT	1.264	1.002–1.593	.048
Tobacco use	1.669	1.349–2.065	.000
Diabetes FH	0.745	0.583–0.953	.019

**Figure 2:**
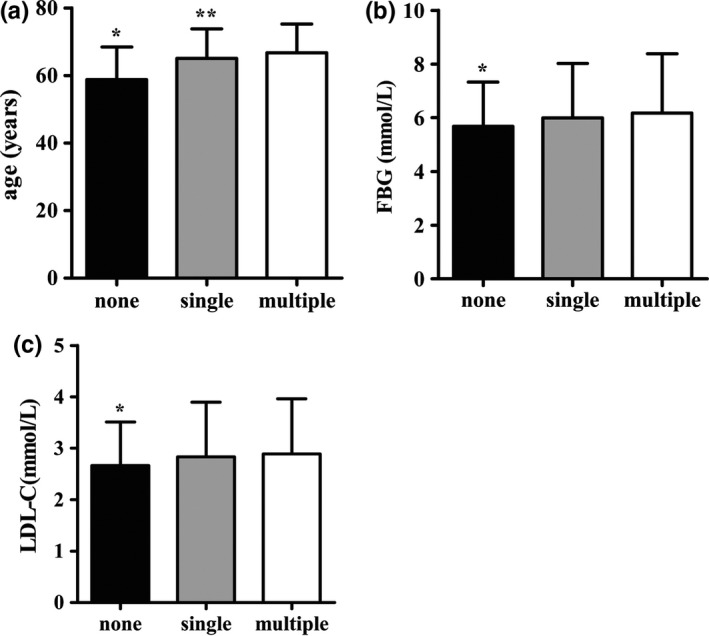
Distribution of age, FBG, LDL‐C in patients without CP and patients with single or multiple CPs. (a) shows the age of different subgroups, there are significant differences in the N‐CP subgroup compared with CP sub‐s (*p =* *.000) and CP sub‐m (*p =* *.000), and a difference is detected between CP sub‐s and CP sub‐m (**p =* *.020). (b) shows the FBG level of different subgroups, there are significant differences in the N‐CP subgroup compared with CP sub‐s (*p =* *.020) and CP sub‐m (*p =* *.000). (c) shows the LDL‐C level of different subgroups, there are significant differences in the N‐CP subgroup compared with CP sub‐s (*p =* *.011) and CP sub‐m (*p =* *.000). Abbreviation: sub‐s, single subgroup; sub‐m, multiple subgroup

**Figure 3:**
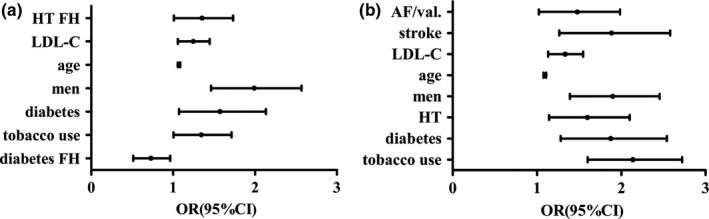
Association between CP number and risk factors. (a) shows the risk factors of single CP are tobacco use, diabetes, male gender, age, high LDL‐C level and family history of hypertension, family history of diabetes is protective. (b) shows the risk factors of multiple CPs are tobacco use, diabetes, hypertension, male gender, age, high LDL‐C level, stroke and AF or valvulopathy

Second, the participants were grouped based on the number of CPs, the distribution of demographic, and clinical data as shown in Table 4. Age, FBG, TC, LDL‐C levels, gender, presence of stroke, hypertension, hyperlipidemia, diabetes, AF or valvulopathy, tobacco use, alcohol use, family history of CHD, hypertension and diabetes, as well as the frequency of eating vegetables and fruits were significantly different among the three subgroups (all *p *<* *.05). Figure 2 shows the distribution of age, FBG, and LDL‐C of the different subgroups. The multivariate logistic regression analysis showed that compared with the N‐CP subgroup, the risk factors of a single CP were tobacco use, diabetes, male gender, age, high LDL‐C levels, and family history of hypertension. However, a family history of diabetes was associated with lower risk.The risk factors of multiple CPs were tobacco use, diabetes, hypertension, male gender, age, high LDL‐C level, stroke and AF or valvulopathy (Table 5, Figure 3).

**Table 4:** Demographic characteristics of the participants, based on the number of CP

	None	Single	Multiple	*p*
Total, *n* (%)	1238 (61.1)	379 (18.7)	408 (20.1)	–
Age (years)	58.7 ± 9.7	65.1 ± 8.8	66.7 ± 8.5	.000
BMI (kg/m^2^)	25.6 ± 3.8	25.3 ± 3.5	25.5 ± 3.2	.280
WHR	0.9 ± 0.1	0.9 ± 0.1	0.9 ± 0.1	.716
FBG (mmol/L)	5.7 ± 1.6	6.0 ± 2.0	6.2 ± 2.2	.000
TG (mmol/L)	2.0 ± 1.4	2.0 ± 1.3	2.0 ± 1.4	.825
TC (mmol/L)	4.8 ± 1.2	5.0 ± 1.4	5.0 ± 1.3	.026
HDL‐C (mmol/L)	1.5 ± 0.5	1.6 ± 0.5	1.5 ± 0.6	.453
LDL‐C (mmol/L)	2.7 ± 0.9	2.8 ± 1.1	2.9 ± 1.1	.000
Men (%)	446 (36.0)	198 (52.2)	218 (53.4)	.000
Women (%)	792 (64.0)	181 (47.8)	190 (46.6)	
Stroke (%)	112 (9.0)	50 (13.2)	72 (17.6)	.000
TIA (%)	159 (12.8)	50 (13.2)	47 (11.5)	.735
HT (%)	881 (71.2)	283 (74.7)	328 (80.4)	.001
HLP (%)	809 (65.3)	275 (72.6)	295 (72.3)	.004
Diabetes (%)	209 (16.9)	92 (24.3)	112 (27.5)	.000
AF/val. (%)	159 (12.8)	62 (16.4)	76 (18.6)	.010
Tobacco use (%)	390 (31.5)	145 (38.3)	195 (47.8)	.000
Alcohol use (%)	281 (22.7)	107 (28.2)	116 (28.4)	.017
PE (%)	562 (45.4)	182 (48.0)	211 (51.7)	.080
Overweight/obesity (%)	548 (44.3)	149 (39.3)	160 (39.2)	.085
Stroke FH (%)	478 (38.6)	135 (35.6)	144 (35.3)	.357
CHD FH (%)	451 (36.4)	118 (31.1)	120 (29.4)	.014
HT FH (%)	766 (61.9)	231 (60.9)	210 (51.5)	.001
Diabetes FH (%)	342 (27.6)	80 (21.1)	83 (20.3)	.002
HLP FH (%)	310 (25.0)	79 (20.8)	95 (23.3)	.233
Vegetables (%)	1075 (86.8)	313 (82.6)	335 (82.1)	.021
Fruits (%)	690 (55.7)	199 (52.5)	184 (45.1)	.001
Milk (%)	281 (22.7)	73 (19.3)	98 (24.0)	.243

**Table 5:** Analysis of the association between CP number and risk factors

	OR	OR (95% CI)	*p*
Single*			
Diabetes FH	0.705	0.515–0.966	.029
Tobacco use	1.313	1.006–1.714	.045
Diabetes	1.513	1.073–2.135	.018
Men	1.940	1.464–2.570	.000
Age	1.073	1.058–1.088	.000
LDL‐C	1.237	1.058–1.446	.008
HT FH	1.322	1.010–1.732	.042
Multiple*			
Tobacco use	2.087	1.599–2.723	.000
Diabetes	1.803	1.280–2.541	.001
HT	1.549	1.144–2.098	.005
Men	1.848	1.391–2.454	.000
Age	1.093	1.078–1.109	.000
LDL‐C	1.323	1.131–1.548	.000
Stroke	1.805	1.264–2.579	.001
AF/val.	1.424	1.021–1.985	.037

*Compared with the N‐CP subgroup.

**Figure 4:**
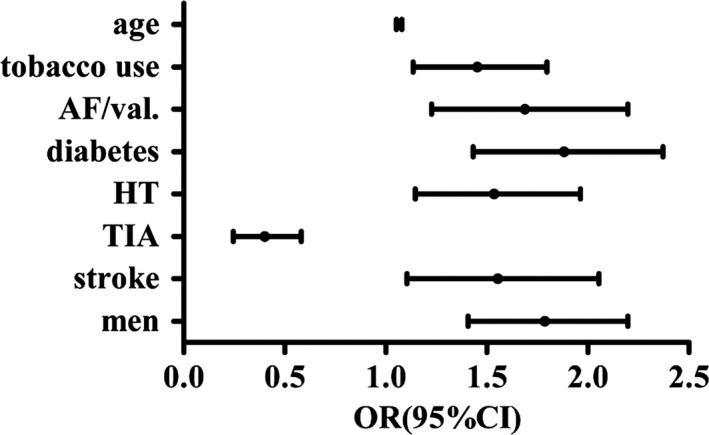
The risk factors of CCAIMT. Figure 4 shows the risk factors of CCAIMT are male gender, stroke, hypertension, diabetes, AF or valvulopathy, tobacco use and age, history of TIA is protective

Third, all of the participants were divided into CCAIMT and N‐CCAIMT groups based on the presence of CCAIMT. Overall, the presence of CCAIMT was 54.1% in men and 45.9% in women (*p *<* *.05). Age, FBG level, presence of stroke, hypertension, diabetes, AF or valvulopathy, tobacco use, and frequency of drinking milk were higher in the CCAIMT group than the N‐CCAIMT group, while the presence of TIA, and frequency of eating vegetables and fruit were higher in the N‐CCAIMT group than the CCAIMT group (all *p *<* *.05; Table 6). The multivariate logistic regression analysis showed that male gender, stroke, hypertension, diabetes, AF or valvulopathy, tobacco use and age were risk factors of CCAIMT, but TIA was possibly associated with a lower risk (Table 7, Figure 4).

**Table 6:** Demographic characteristics of the participants, based on the presence of CCAIMT

	CCAIMT	N‐CCAIMT
Total, *n* (%)	503 (24.8)	1522 (75.2)
Age (years)	66.2 ± 8.6*	60.0 ± 9.9
BMI (kg/m^2^)	25.6 ± 3.4	25.5 ± 3.8
WHR	0.9 ± 0.1	0.9 ± 0.1
FBG (mmol/L)	6.2 ± 2.1*	5.7 ± 1.8
TG (mmol/L)	2.0 ± 1.4	2.0 ± 1.4
TC (mmol/L)	4.9 ± 1.4	4.9 ± 1.2
HDL‐C (mmol/L)	1.6 ± 0.6	1.5 ± 0.5
LDL‐C (mmol/L)	2.7 ± 1.0	2.7 ± 0.9
Men (%)	272 (54.1)*	590 (38.8)
Women (%)	231 (45.9)*	932 (61.2)
Stroke (%)	83 (16.5)*	151 (9.9)
TIA (%)	29 (5.8)*	227 (14.9)
HT (%)	407 (80.9)*	1085 (71.3)
HLP (%)	353 (70.2)	1026 (67.4)
Diabetes (%)	146 (29.0)*	267 (17.5)
AF/val. (%)	97 (19.3)*	200 (13.1)
Tobacco use (%)	212 (42.1)*	518 (34.0)
Alcohol use (%)	133 (26.4)	371 (24.4)
PE (%)	267 (53.1)	839 (55.1)
Overweight/obesity (%)	198 (39.4)	659 (43.3)
Stroke FH (%)	190 (37.8)	561 (36.9)
CHD FH (%)	162 (32.2)	527 (34.6)
HT FH (%)	249 (49.5)	821 (53.9)
Diabetes FH (%)	114 (22.7)	391 (25.7)
HLP FH (%)	125 (24.9)	359 (23.6)
Vegetables (%)	416 (82.7)*	1329 (87.3)
Fruits (%)	251 (49.9)*	839 (55.1)
Milk (%)	139 (27.6)*	316 (20.8)

*p <* *.05 vs N‐CCAIMT group.

**Table 7:** The risk factors of CCAIMT

	OR	OR(95% CI)	*p*
Men	1.757	1.406–2.196	.000
Stroke	1.506	1.105–2.053	.010
TIA	0.379	0.246–0.583	.000
HT	1.500	1.146–1.962	.003
Diabetes	1.843	1.431–2.372	.000
AF/val.	1.641	1.227–2.196	.001
Tobacco use	1.429	1.135–1.798	.002
Age	1.065	1.053–1.078	.000

**Figure 5:**
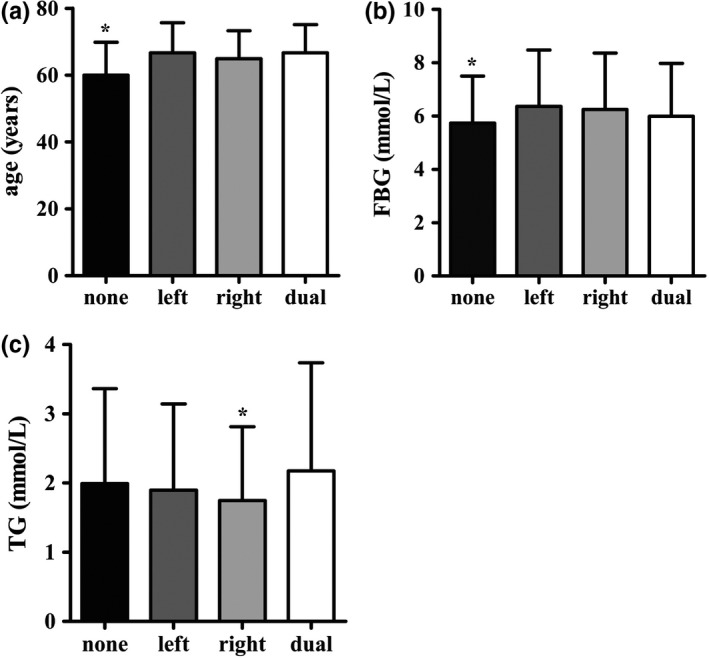
Distribution of age, FBG, TG in patients without CCAIMT and patients with CCAIMT on different side. (a) shows the age of different subgroups, there are significant differences in the N‐CCAIMT subgroup compared with CCAIMT sub‐l, sub‐r, sub‐d (*p =* *.000). (b) shows the FBG level of different subgroups, there are significant differences in the N‐CCAIMT subgroup compared with CCAIMT sub‐l (*p =* *.002) and sub‐r (*p =* *.048). (c) shows the TG level of different subgroups, there is significant difference between CCAIMT sub‐r and sub‐d (*p =* *.018). Abbreviation: sub‐l, left side subgroup; sub‐r, right side subgroup; sub‐d, dual sides subgroup

**Figure 6:**
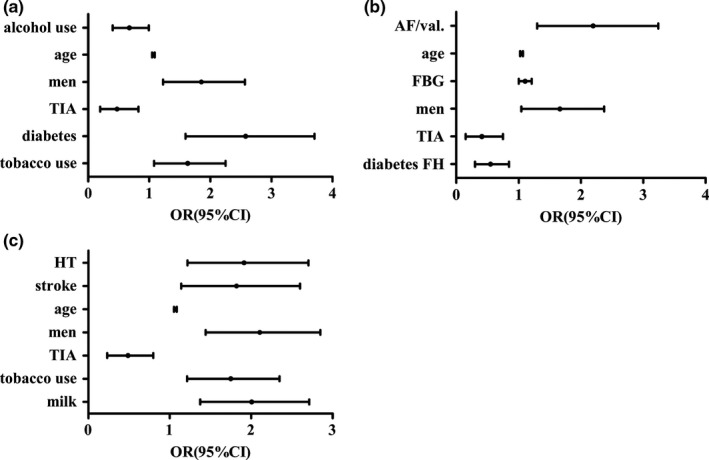
Association between CCAIMT side and risk factors. (a) shows the risk factors of left CCAIMT are tobacco use, diabetes, male gender, age, history of TIA and alcohol use are protective. (b) shows the risk factors of right CCAIMT are male gender, high FBG level, age and AF or valvulopathy, history of TIA and family history of diabetes are protective. (c) shows the risk factors of dual CCAIMT are high frequency of drinking milk, tobacco use, male gender, age, stroke, hypertension, history of TIA is protective

Finally, Table 8 shows the distribution of the demographic and clinical data of all of the participants, grouped according to the side of the CCAIMT. Age, FBG, TG levels, gender, presence of stroke, TIA, hypertension, diabetes, AF or valvulopathy, tobacco and alcohol use, and frequency of eating vegetables and drinking milk were significantly different among the four subgroups (all *p *<* *.05). Figure 5 shows the distribution of age, FBG, and TG of the different subgroups. Multivariate logistic regression analysis showed that compared with the N‐CCAIMT subgroup, the risk factors of left CCAIMT were tobacco use, diabetes, male gender, and age, while the history of TIA and alcohol use was protective. The risk factors of right CCAIMT were male gender, high FBG level, age, AF or valvulopathy, and the history of TIA, whereas a family history of diabetes was associated with a lower risk. The risk factors of dual CCAIMT were high frequency of drinking milk, tobacco use, male gender, age, stroke and hypertension, while a history of TIA was associated with lower risk (Table 9, Figure 6).

**Table 8:** Demographic characteristics of the participants, based on the side of CCAIMT

	None	Left	Right	Dual	*p*
Total, *n* (%)	1522 (75.2)	163 (8.0)	128 (6.3)	212 (10.5)	–
Age (years)	60.0 ± 9.9	66.7 ± 8.9	64.9 ± 8.3	66.7 ± 8.5	.000
BMI (kg/m^2^)	25.5 ± 3.8	25.8 ± 3.5	25.4 ± 3.4	25.6 ± 3.3	.842
WHR	0.9 ± 0.1	0.9 ± 0.1	0.9 ± 0.1	0.9 ± 0.7	.338
FBG (mmol/L)	5.7 ± 1.8	6.4 ± 2.1	6.3 ± 2.1	6.0 ± 2.0	.000
TG (mmol/L)	2.0 ± 1.4	1.9 ± 1.2	1.8 ± 1.1	2.2 ± 1.6	.034
TC (mmol/L)	4.9 ± 1.2	4.9 ± 1.4	4.9 ± 1.2	4.9 ± 1.5	.969
HDL‐C (mmol/L)	1.5 ± 0.5	1.6 ± 0.5	1.5 ± 0.4	1.6 ± 0.7	.128
LDL‐C (mmol/L)	2.7 ± 0.9	2.8 ± 1.0	2.6 ± 0.8	2.8 ± 1.2	.608
Men (%)	590 (38.8)	83 (50.9)	62 (48.4)	127 (59.9)	.000
Women (%)	932 (61.2)	80 (49.1)	66 (51.6)	85 (40.1)	
Stroke (%)	151 (9.9)	26 (16.0)	18 (14.1)	39 (18.4)	.001
TIA (%)	227 (14.9)	9 (5.5)	7 (5.5)	13 (6.1)	.000
HT (%)	1085 (71.3)	130 (79.8)	100 (78.1)	177 (83.5)	.000
HLP (%)	1026 (67.4)	108 (66.3)	90 (70.3)	155 (73.1)	.344
Diabetes (%)	267 (17.5)	60 (36.8)	34 (26.6)	52 (24.5)	.000
AF/val. (%)	200 (13.1)	29 (17.8)	30 (23.4)	38 (17.9)	.003
Tobacco use (%)	518 (34.0)	68 (41.7)	46 (35.9)	98 (46.2)	.002
Alcohol use (%)	371 (24.4)	33 (20.2)	28 (21.9)	72 (34.0)	.007
PE (%)	839 (55.1)	75 (46.0)	74 (57.8)	118 (55.7)	.130
Overweight/obesity (%)	659 (43.3)	68 (41.7)	49 (38.3)	81 (38.2)	.400
Stroke FH (%)	561 (36.9)	65 (39.9)	46 (35.9)	79 (37.3)	.884
CHD FH (%)	527 (34.6)	52 (31.9)	34 (26.6)	76 (35.8)	.255
HT FH (%)	821 (53.9)	90 (55.2)	59 (46.1)	100 (47.2)	.103
Diabetes FH (%)	391 (25.7)	42 (25.8)	20 (15.6)	52 (24.5)	.091
HLP FH (%)	359 (23.6)	39 (23.9)	31 (24.2)	55 (25.9)	.902
Vegetables (%)	1329 (87.3)	133 (81.6)	112 (87.5)	171 (80.7)	.017
Fruits (%)	839 (55.1)	76 (46.6)	69 (53.9)	106 (50.0)	.128
Milk (%)	316 (20.8)	40 (24.5)	26 (20.3)	73 (34.4)	.000

**Table 9:** Analysis of the association between CCAIMT side and risk factors

	OR	OR (95% CI)	*p*
Left*			
Tobacco use	1.559	1.079–2.251	.018
Diabetes	2.432	1.596–3.706	.000
TIA	0.402	0.196–0.823	.013
Men	1.773	1.225–2.568	.002
Age	1.068	1.048–1.088	.000
Alcohol use	0.632	0.402–0.993	.046
Right*			
Diabetes FH	0.504	0.300–0.846	.010
TIA	0.336	0.151–0.748	.008
Men	1.574	1.043–2.373	.031
FBG	1.102	1.002–1.211	.045
Age	1.047	1.026–1.068	.000
AF/val.	2.051	1.297–3.241	.002
Dual*			
Milk	1.933	1.376–2.715	.000
Tobacco use	1.687	1.212–2.350	.002
TIA	0.432	0.234–0.799	.007
Men	2.026	1.440–2.850	.000
Age	1.069	1.052–1.087	.000
Stroke	1.723	1.142–2.600	.009
HT	1.815	1.218–2.704	.003

*Compared with the N‐CCAIMT subgroup.

HT, hypertension; TIA, transient ischemic attack; FBG, fasting blood glucose.

## 4 DISCUSSION

This cross‐sectional study described the presence and risk factors of CP and CCAIMT in a high‐stroke‐risk population. The overall CP presence was 38.9% and overall CCAIMT presence was 24.8%. In addition, age, male gender, high FBG and LDL‐C levels, stroke, diabetes, hypertension, and tobacco use were risk factors of CP. We also determined an association between CP numbers and risk factors. This study also found the risk factors of CCAIMT were male gender, age, stroke, hypertension, diabetes, AF or valvulopathy and tobacco use. We also showed an association between the side of the CCAIMT and risk factors.

In the Northern Manhattan Study (NOMAS), an urban, multiethnic population‐based study, CPs were present in 58% of the participants (Yang et al., 2015). Another study showed that the presence of CPs was 60.3%, the mean intima‐media thickness of the carotid artery was 0.68 mm, and the maximal intima‐media thickness was 1.07 mm among the middle‐aged and elderly population in Beijing, China (Wang et al., 2010). However, no studies have shown the exact prevalence of CCAIMT. In this study, the presence of CP and CCAIMT were 38.9% and 24.8% respectively.

The risk factors of CPs or CIMT have been discussed in several population‐based cohort studies. These studies have explored the association between traditional cardiovascular or cerebrovascular risk factors, such as serum lipid parameter, age, gender, hypertension, diabetes, smoking and CPs or CIMT. It was agreed consistently that these risk factors increased the presence of CPs and CIMT (Calmarza, Trejo, Lapresta, & Lopez, 2015; Panayiotou et al., 2013; Tiozzo et al., 2014; Touboul et al., 2011; Yang et al., 2014; Zhan et al., 2016). In this study, the independent risk factors of CP were male gender, age, high FBG and LDL‐C levels, stroke, diabetes, hypertension and smoking; the risk factors of CCAIMT were male gender, age, stroke, hypertension, diabetes, AF or valvulopathy and smoking, whereas alcohol use was associated with a lower risk. These findings were similar to the previous studies. However, in the present study, TIA and a family history of diabetes were associated with lower risk, which is different from the conventional theory. We noticed a phenomenon in the process of interviewing, where some residents said that they would pay more attention to their blood glucose levels than others from a young age, because of a family history of diabetes. It may suggest that early detection and prevention of risk factors helps to delay the development of carotid atherosclerosis in the participants with a family history of diabetes. The reason for the protective value of TIA history shown in this study may be due to confusion of the concept when residents self‐reported their previous history of TIA.

People with different number of CPs may also have differences in their demographic characteristics. In the present study, the risk factors of a single CP were tobacco use, diabetes, male gender, age, high LDL‐C level and a family history of hypertension, while the participants with risk factors of tobacco use, diabetes, hypertension, male gender, older age, high LDL‐C level, stroke and AF or valvulopathy were more likely to have multiple CPs. The Three City Study, a French community‐based cohort study of older adults, divided the participants into three groups according to the number of CPs and revealed that the mean levels of risk factors were increased with the number of CPs (Plichart et al., 2011). Another finding of the Rotterdam Study was a dose‐dependent relationship between the risk of stroke and carotid plaques. The participants with 3–4 plaques had 5‐fold increased risk, participants with 5–6 plaques had 10‐fold increased risk of lacunar infarction, regardless of their locations (Hollander et al., 2002). More risk factors and higher level of risk factors mean higher risk of diseases, so the high‐risk population mentioned above need to modify their risk factors more strictly to avoid the serious consequences of cerebrovascular or cardiovascular diseases.

There were limited studies focused on the differences in risk factors of having CCAIMT on different sides. A previous study enrolled 88 patients with type‐2 diabetes and found that the rate of carotid atherosclerosis development was much higher on the left side than on the right side (Li, Liu, Du, & Luo, 2016). In Greek adolescents, a similar conclusion was found, where CIMT of the left carotid artery was higher and more closely associated with cardiovascular risk factors (Kollias et al., 2013). Hypertensive subjects had higher values of CCAIMT on the left side (Rodriguez Hernandez et al., 2003). The reason of this unilateral dominance phenomenon is still not clear, but a possible mechanism is the different anatomical origins of carotid arteries. One study analyzed the carotid ultrasound results of 532 subjects from six medical centers, and found that the hemodynamic and biochemical changes affected bilateral CCAIMT differently; the left and right CCAIMT correlated with blood biochemical indices and hemodynamic parameters respectively (Luo, Yang, Cao, & Li, 2011). In this study, the presence of left CCAIMT (8.0%) was higher than right CCAIMT (6.3%); furthermore, subjects with diabetes were more likely to have left CCAIMT. This is in accord with the above‐mentioned results. When compared with the N‐CCAIMT subgroup, the participants who were male and older in age, with high FBG level and AF or valvulopathy were more likely to have right CCAIMT, whereas the participants with risk factors of older age, male gender, tobacco use, hypertension, stroke, and high frequency of drinking milk were prone to suffer CCAIMT on dual sides. This finding also gave us a guideline for risk factors modifications among the high‐risk population.

In conclusion, this cross‐sectional population‐based study revealed the risk factors of CPs and CCAIMT. The subjects with different risk factors also have different CP numbers and side of CCAIMT. For patients with different cardiovascular or cerebrovascular risk factors, we would emphasize the control strategy differently.

There were limitations in this study that we need to mention. We did not analyze the CPs and CCAIMT quantitatively. A more detailed study could be helpful to determine a quantitative association between the risk factors and carotid atherosclerosis.

## CONFLICT OF INTEREST

There is no conflict of interest.
